# Tonsil neuroendocrine carcinoma concurrent with hepatocellular carcinoma: A case report

**DOI:** 10.3892/ol.2014.2309

**Published:** 2014-07-03

**Authors:** YAN YUAN, QING-FENG ZOU, XIAO-YE HU, MEI-YUAN LIU

**Affiliations:** Cancer Center, Guangzhou Medical University, Guangzhou, Guangdong 510095, P.R. China

**Keywords:** neuroendocrine carcinoma, hepatocellular carcinoma, tonsil, head and neck

## Abstract

The majority of neuroendocrine tumors appear to be sporadic. Neuroendocrine carcinoma (NEC) typically arises in pancreatic, parathyroid and adrenal glands, but rarely arises in salivary glands. NEC of the tonsil is a rare type of tumor and the concurrent presentation of hepatocellular carcinoma (HCC) is considered to be more uncommon. There are few case reports of NEC of the tonsil in the literature and to date no studies have been conducted to establish its optimal management. The current study presents a case of a 72-year-old male who presented with left neck and tonsil tumors. A biopsy from the tonsil revealed a NEC, and computed tomography showed liver cirrhosis, multiple liver cancers and portal vein thrombosis, as well as metastasis to the hilar, abdomen and retroperitoneum. Histological examination of the hepatic revealed primary HCC. To the best of our knowledge, this is a condition that has not previously been reported.

## Introduction

It is hypothesized that neuroendocrine carcinoma (NEC) arise from cells that are involved in the diffuse endocrine system (1). The biological activity and prognosis of NEC are associated with various factors, including the tumor site, histological type and degree of differentiation. A number of studies have confirmed that poorly differentiated NEC is associated with an aggressive clinical course and poor prognosis (2,3). Numerous patients with NEC develop hepatic metastasis (4), which must be distinguished from primary hepatocellular carcinoma (HCC). HCC is one of the most common types of tumor worldwide and one of the most malignant. Furthermore, patients with chronic hepatitis B viral infection or alcoholic cirrhosis are at increased risk of developing HCC (5). In the present case, the patient was diagnosed with primary HCC within a few months of being diagnosed with a tonsil NEC. Such NEC of the tonsil tend to be aggressive and are associated with a poor prognosis. This report describes one case of tonsil neuroendocrine carcinoma concurrent with hepatocellular carcinoma. Patient provided written informed consent.

## Case report

A 72-year-old male was admitted to the Gaozhou People’s Hospital (Gaozhou, China) in March 2013 with a four-month history of a left neck tumor. Fine-needle aspiration of the left tonsil mass had been performed at two other hospitals and was identified to be positive for a small-cell malignancy or NEC. The patient was referred to The Affiliated Tumor Hospital of Guangzhou Medical University (Guangzhou, China) for further treatment. The patient’s medical history included hypertension, which had been present for six years with intermittent use of oral antihypertensive agents. In addition, the patient was infected with the hepatitis B virus (HBV) and had suffered with liver cirrhosis for 15 years. The patient was a non-smoker, however, occasionally consumed alcohol. The physical examination was notable for a solitary, left, level II tonsil mass (size, 3.6×2.0 cm), according to the following chinese antiadoncus clinical classification system: I, Tonsil enlargement which does not exceed the pharyngeal arch palate; II, tonsil enlargement exceeds the pharyngeal arch palate, but does not exceed the midline of the posterior pharyngeal wall; and III, tonsil enlargement exceeds the midline of the posterior pharyngeal wall (6). There were numerous enlarged nodes on each side of the neck; the largest node was 2.2×1.1 cm. The laboratory tests were abnormal; the α-fetoprotein (AFP) level had increased to 9.2×10^4^ ng/ml (normal range, 0–25 ng/ml), and carbohydrate antigen 19-9 (normal range, 0–37 U/ml), cancer antigen 125 (normal range, 0–35 kU/l) and neuron specific enolase (NSE; normal range, 0–12.5 U/ml) were all increasing. In addition, the level of HBV-DNA was 1.62×10^7^ IU/ml (normal range, 0–50 IU/ml). However, alanine aminotransferase (normal range, 10–40 IU/l), aspartate aminotransferase (normal range, 10–40 IU/l) and bilirubin (normal range, 3.4–17.1 μmol/l) were all observed to be within the normal ranges.

**Figure 1:**
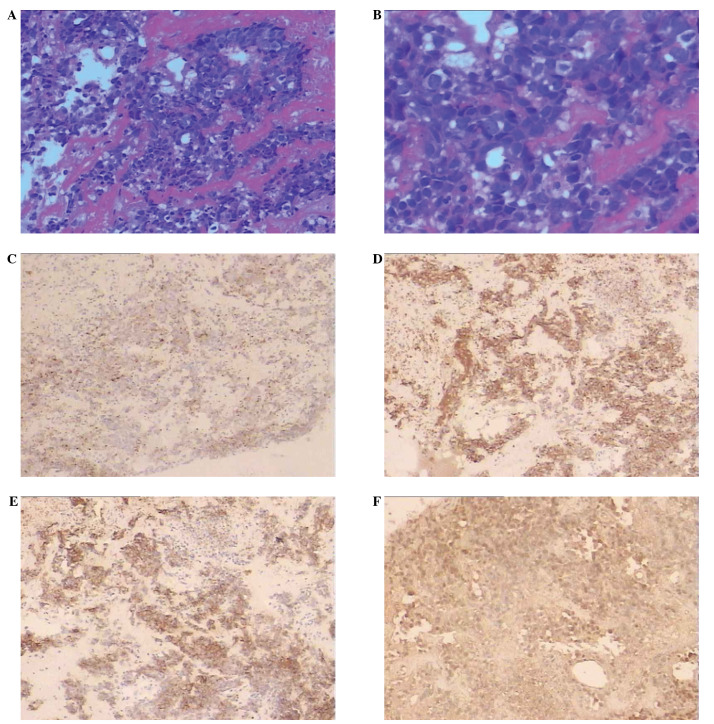
(A and B) Microscopic observations of the tonsil neuroendocrine carcinoma (NEC). The NEC comprised of small and middle-sized cells, which demonstrate granular to homogeneous-stained hyperchromatic nucleis that were pathologically identified as mitotic (stain, hematoxylin and eosin; A: magnification, ×100; B: magnification, ×200). (C–F) Immunohistochemistry identified the NEC cells as positive for chromogranin A, synaptophysin, cluster of differentiation 56 and α-fetoprotein in the cytoplasm (labeled avidin-biotin staining; magnification, ×100).

**Figure 2:**
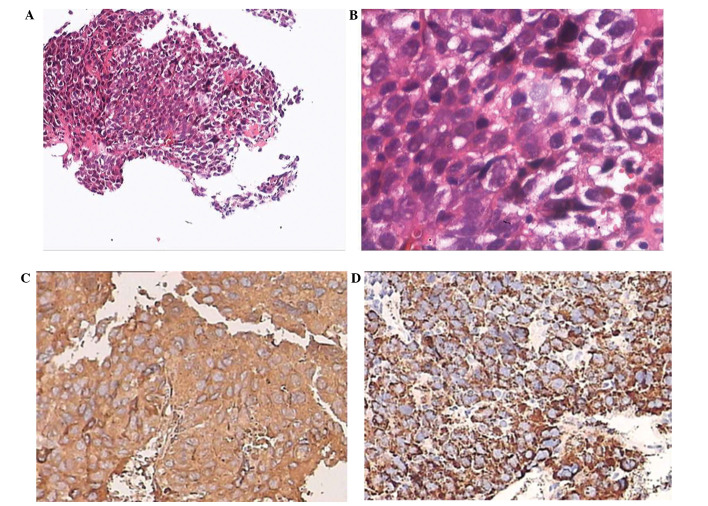
(A and B) Microscopic observations of the poorly differentiated hepatocellular carcinoma (HCC). The HCC tissue comprised of tumor cells of varying sizes. The cytoplasmic boundaries are clear, with red staining demonstrating the large irregularly-shaped tumor cells scattered in the nucleaus that were pathologically identified as mitotic (stain, hematoxylin and eosin; A: magnification, ×100; B: magnification, ×200). (C and D) Immunohistochemistry and histochemical analysis of the primary HCC demonstrate the tumor cells to be positive for Hepatocyte Specific Antigen antibody and α-fetoprotein in the cytoplasm. The liver tissue appears to be denatured and fibrous tissue proliferation is apparent in the periportal (labeled avidin-biotin staining; magnification, ×200).

Contrast-enhanced magnetic resonance imaging of the oropharyngeal airway revealed a 3.6-cm mass of the left palate and there were numerous enlarged nodes on each side of the neck, with the largest measuring 2.2×1.1 cm. A computed tomography (CT) scan of the chest and abdomen demonstrated liver cirrhosis, multiple liver tumors and portal vein thrombosis, as well as metastasis to the hilar, abdomen and retroperineum. Histologic examination of the tonsil revealed an NEC (Fig. 1A and B). Immunohistochemistry was positive for chromogranin A (CgA), synaptophysin (Syn), cluster of differentiation (CD)56, AFP, and some hepatocytes and negative for cytokeratin (CK), p63, melan-A, CD3, CD10, CD20, CD30, anaplastic lymphoma kinase, CAM5.2, *PAX-5* and CD43. The cell growth index was 80%. Various tumors located in the right hepatic posterior lobe and right adrenal gland were identified by color Doppler ultrasound. The largest liver tumor measured 3.3×3.1 cm and was located in the right lobe. Histologic examination of the liver revealed a poorly differentiated primary HCC (Fig. 2A and B). Immunohistochemistry was positive for Hepatocyte Specific Antigen antibody (Hep Par-1), AFP, and a small quantity of CK and was negative for CgA, Syn, CD56 and inhibin. The cell growth index was 50%. A biopsy of the right adrenal tumor was not performed, as it was located between the right hepatic lobe and the right kidney which is unsuitable for biopsy. The size of the oval-shaped tumor was observed via color Doppler ultrasound to be ~5.3×4.0 cm. The patient was diagnosed with NEC of the right tonsil with metastatic disease to the neck, a poorly differentiated HCC, right adrenal metastatic tumor and level 3 hypertension, and was considered to be at a particularly high-risk stage. The patient received one cycle of palliative chemotherapy lasting two days with a cycle duratino of 21 days with carboplatin (0.5 g) and etoposide (EP; 0.2 g) on days one to two. The masses continued to grow and the size of the hepatic tumor increased to 8.0×6.2 cm. Treatment with carboplatin and EP failed to inhibit the disease progression, and the tonsil carcinoma became larger and was almost completely blocking the oropharyngeal airway. Radiotherapy was administered with the aim of controlling the growth of the tonsil tumor. However, the patient abandoned the treatment and succumbed due to asphyxia two days after leaving The Cancer Center of Guangzhou Medical University (Guangzhou, China).

## Discussion

NEC are a heterogeneous family of neoplasms that possess a broad spectrum of types of histomorphology, tissue origins and clinical behaviors (7). NEC generally exhibit slow growth, however, prognosis is dependent on the tumor site, histological type, degree of differentiation, mitotic rate, Ki-67 proliferative index, tumor size, depth, location and the presence of lymph node or liver metastases (8). The recent WHO classification (year 2000) defines tumors, on the basis of histopathological and biological characteristics, into well-differentiated NEC (benign or uncertain malignancy), well-differentiated NEC (low-grade malignancy), poorly differentiated NEC (high-grade malignancy) and mixed tumors (9).

NEC of the head and neck are rare and, therefore, case reports are only sporadically observed in the literature. A previous study reported metastatic NEC to the head and neck in certain primary lung or breast NEC patients (7). In addition, a previous study described a case of tonsillar metastasis from a primary early-stage large cell NEC of the lung (10). In the current patient, the origin of the tumor was hypothesized to be the tonsil as no tumors were observed in the lung by radiological examination and there are currently few reports concerning the metastasis of a tonsil NEC from a primary liver NEC. Furthermore, the microscopic findings, large size of the tumor and the enlarged neck lymph nodes supported this hypothesis. Thus, primary NEC arising from the tonsil was considered to be the most appropriate diagnosis. To the best of our knowledge, this may be the first report of a primary tonsil NEC.

The majority of previous reports describe cases of tumors with a laryngeal origin where the tumors are predominantly moderately differentiated (11). However, the pathology slides of the patient in the present study failed to provide the level of differentiation. In addition, as the standard therapy for tonsil NEC has not yet been established, the treatment was conducted with a strategy that is commonly used for laryngeal NEC. Barker *et al* (12) investigated 23 adults with nonsinonasal NEC (NSNEC) of the head and neck, and recommended the treatment strategy of sequential chemotherapy and radiation. In this study, there were 19 cases of small-cell undifferentiated carcinomas and the use of combination chemotherapy approximately doubled the two-year overall survival and disease-free survival rates, and reduced the two-year rate of distant metastasis by half. As NSNEC was found to be highly responsive to cisplatin/EP combination chemotherapy the induction chemotherapy strategy was adopted in the present case. However the outcome was not positive and the efficacy of radiotherapy remains unknown. Further studies are required to elucidate an optimal treatment strategy for tonsil NEC.

A total of 50–95% of patients with NEC develop liver metastases and 80% of patients exhibiting advanced liver disease succumb within five years of diagnosis (13). Furthermore, certain cases of primary hepatic NEC have been described in previous studies (14,15). Therefore, a careful clinical evaluation is essential to distinguish the extrahepatic origins of tumors; either primary hepatic NEC or HCC (as in the present case). Kaya *et al* (15) reported one case of primary NEC of the liver in 2001. In this case, the tumor cells were positively stained for CgA and Syn (the immunological markers for tumors derived from the neuroendocrine system) and negatively stained for AFP. In contrast to HCC, hepatic NEC has not previously been associated with liver cirrhosis. A review of the literature identified that the immunohistochemical characteristics of HCC include positivity for the neurosecretory markers, CgA, Syn and NSE, and negativity for Hep Par-1 (OCH1E5), AFP, thyroid transcription factor-1, *CDX2* and leukocyte common antigen. A percutaneous biopsy of the liver mass was performed in the present case and immunohistochemistry revealed a poorly differentiated HCC.

The incidence of two types of cancer presenting in one patient is rare; however, ~20% of patients with NEC develop secondary cancers (8). Combined primary NEC and HCC of the liver in a 65-year-old male patient was reported in 2009 by Yang *et al* (14). It was proposed that the NEC originated from a poorly differentiated tumor clone of an HCC that had undergone neuroendocrine differentiation. Ki-67 and p53 expression were identified to be higher in the NEC compared with that in the HCC. Furthermore, HCC is one of the leading causes of cancer-associated mortality. HCC progress so rapidly that the majority of patients are diagnosed with locally advanced or distant metastasis; therefore, the resulting treatment efficacy and prognosis is poor. The tumor exhibited a more aggressive clinical course in accordance with being an NEC, rather than a conventional HCC, and the patient succumbed due to multiple recurrent tumors and metastases within a year after surgery. In the present case, the Ki-67 proliferative index in the tonsil NEC was identified to be higher than that in the HCC. Considering the aggressive biological behavior of the NEC, the tonsil NEC was initially treated using chemotherapy. However, the patient deteriorated and succumbed within two months of the diagnosis.

In conclusion, NEC of the head and neck is uncommon and has rarely been described in the tonsil. With regard to NEC, the prognosis of this type of tumor appears to be poorer when it is located in the tonsil compared with in other sites of the head and neck. In addition, it was identified that tonsil NEC is not sensitive to a chemotherapy regimen that contained carboplatin and EP. Thus, the optimal treatment for NEC of the tonsil remains unclear.
